# Ultrafast Photofragmentation of Ln(hfac)_3_ with a Proposed Mechanism for forming High Mass Fluorinated Products

**DOI:** 10.1038/s41598-020-64015-2

**Published:** 2020-04-27

**Authors:** Jiangchao Chen, Xi Xing, Roberto Rey-de-Castro, Herschel Rabitz

**Affiliations:** Department of Chemistry, Princeton University, Princeton, New Jersey 08544 USA

**Keywords:** Photochemistry, Physical chemistry

## Abstract

The photo-induced dissociative-ionization of lanthanide complexes Ln(hfac)_3_ (Ln = Pr, Er, Yb) is studied using intense ultrafast transform limited (TL) and linearly chirped laser pulses in a time-of-flight (TOF) mass spectrometry setup. Various fluorine and Ln-containing high-mass fragments were observed in this experiment, including the molecular parent ion, which have not been seen with previous studies relying on relatively long-duration laser pulses (i.e., ns or longer). These new high-mass observations provide important formerly missing information for deducing a set of photo-fragmentation mechanistic pathways for Ln(hfac)_3_. An overall ultrafast control mechanism is proposed by combining insights from earlier studies and the fragments observed in this research to result in three main distinct photo-fragmentation processes: (a) ligand-metal charge transfer, (b) CF_3_ elimination, and (c) C-C bond rotation processes. We conclude that ultrafast dissociative-ionization could be a promising technique for generating high-mass fragments for potential use in material science applications.

## Introduction

There are potentially wide-ranging applications of lanthanide fluorides in the field of materials science, where the molecules or specific fragments from them can form inorganic matrices serving as phosphors due to their low degree of phonon energy coupling^1^. For example, efficient energy transfer from Yb to Er has been obtained in a NaYF_4_ matrix to form highly luminescent near infrared-to-visible up-conversion in the nanocrystals β-NaYF_4_:Er,Yb^2^. The β-diketonate complexes, such as Ln(thd)_3_ (thd-=2,2,6,6-tetramethyl-3,5-heptanedionate), are traditional precursors for thin films of lanthanide materials during metal-organic chemical vapor deposition (MOCVD)^3^. Fluorinated lanthanide complexes, Ln(hfac)_3_ (hfac = hexafluoroacetylacetonate) and Ln(fod)_3_ (fod = 1,1,1,2,2,3,3-heptafluoro-7,7-dimethyl-4,6-octanedionate), are also commonly used as precursors in MOCVD, because of their high thermal stability, volatility^4,5^, and superior mass transport properties^6,7^. Even though Ln(hfac)_3_ and Ln(fod)_3_ are both oxygen-coordinated complexes, they are still excellent candidates for the deposition of lanthanide fluorides upon suitable photofragmentation^7,8^.

Previous gas phase studies have considered the photofragmentation mechanisms of Ln(hfac)_3_ and Ln(fod)_3_, with a propensity for formation of lanthanide fluorides^6,9–11^. The metal fluoride formation is consistent with earlier results by Zink *et al*.^10^ who observed the spectroscopic signature of CrF following Cr(hfac)_3_ photolysis with ns lasers at ~400 nm. They proposed a unimolecular reaction that was initiated by the rotation of the C_α_-C(O) bond bringing the CF_3_ group into the proximity to the metal^10^. A similar rotation-based mechanism was proposed by Condorelli and co-workers as a reasonable means for explaining the production of SrF_2_ from the MOCVD of Sr(hfac)_2_tetraglyme^11^. Pollard and co-workers further observed that the metal fluoride formation was accompanied by the elimination of CO^6^.

In the case of Ln(hfac)_3_ and Ln(fod)_3_, high mass fragments have been rarely observed during experiments that used nanosecond or longer-pulsed lasers as fragmentation sources^9^. Without information about the nature of the high mass fragments, it is difficult to deduce an overall picture of the formation mechanism of lanthanide fluorides.

Intense, ultrashort laser pulses are capable of strongly interacting with matter resulting in phenomena such as above threshold ionization^12,13^, high harmonic generation^14–16^, Coulomb explosion^17^, nonadiabatic excitation^18,19^, and even neutron emission^20,21^. Nakashima found that strong molecular fragmentation can occur when the excitation wavelength is resonant with cation electronic levels^22^. For excitation at non-resonant wavelengths, a sufficiently intense short pulse is often necessary to achieve significant parent molecular ionization, while suppressing further molecular fragmentation^22^. In previous studies with ns lasers at 266, 355 and 532 nm as the fragmentation sources, the mass spectrum for Ln(hfac)_3_ shows no peak signal over the noise level for the parent ion or for metal-containing mass fragments heavier than LnF_2_^+^^9^.

In this paper we show that intense femtosecond laser pulses can generate a variety of high mass fragments, including the parent ion. We also show that the formation of fluorine-containing photofragments can be understood by combining information from the prior long pulse experiments with the present ultrafast data. An overall photodissociation mechanism is presented based on the combined experiments to propose three main distinct pathways: (a) ligand-metal charge transfer (LMCT), (b) CF_3_ elimination, and (c) C_α_-C(O) bond rotation. The observation of high mass fragments provides important clues towards elucidating whether metal fluorination occurs in a stepwise mechanism, with one fluorine atom being deposited per ligand. The presence of high-mass fragments may also indicate that two of the three ligands are involved in metal fluorination.

**Figure 1:**
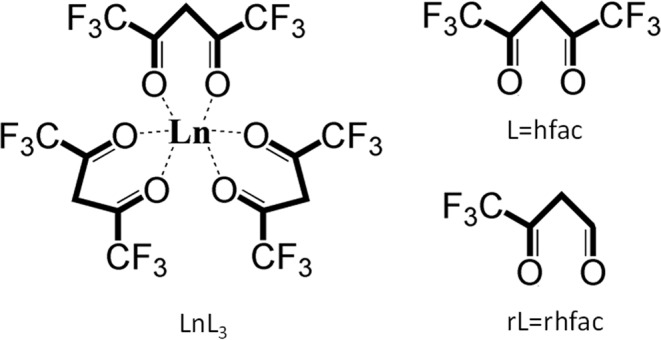
Chemical structure of LnL_3_ and its ligands. (**a**) Chemical structure of LnL_3_; (**b**) Chemical structure of Ligand L, where L stands for hfac; (**c**) Chemical structure of ligand rL, where rL stands for rhfac (i.e., elimination of one CF_3_ group from ligand L) in this illustration.

The paper is organized as follows: Section II describes the experimental setup including the laser system, the means for sample delivery, and the vacuum system. Section III presents the results and a discussion focused on deducing fragmentation pathways from the mass spectra of Ln(hfac)_3_ with transform limited (TL) and linearly chirped fs-laser pulses. Section IV provides a conclusion for the mechanistic understanding of the dissociative ionization studies of Ln(hfac)_3_ with fs laser pulses. In remainder of the paper we will often use simplified notation for the compounds, parent ions and photofragments, such as L = hfac, rL=rhfac (i.e., hfac loss of one CF_3_), Ln=lanthanide elements (i.e., Pr, Er, and Yb in this work). The structure of the compounds associated with the notation above is shown in Fig. 1.

## Experimental Setup

As described in more detail previously^23^ a linear time-of-flight mass spectrometer (Jordan TOF) was used to detect laser induced positively charged fragments. The laser employed in the experiments is a femtosecond Ti:Sapphire laser system consisting of an oscillator and a multi-pass amplifier (KMlab, Dragon) operating at a repetition rate of 3 kHz with pulses of 35 ± 4 fs width at ~785 nm. A pulse shaper with a dual-mask liquid crystal SLM containing 640 pixels (CRI, SLM-640), was used to (1) correct for distortions to generate TL pulses, and (2) perform linearly chirped pulses. The TL and chirped pulses were characterized by standard SHG-FROG^24^. Each TL pulse with 200 ± 20 µJ energy, was then focused with a fused silica lens of f = 20 cm into an optically-accessible vacuum chamber (part of the mass spectrometer), to a spot size of ~50 µm diameter; each pulse is estimated to have a maximum peak intensity of ~3 × 10^14^ W/cm^2^ focused in between the repeller plate (3 kV) and extraction grid (2 kV) of the mass spectrometer.

The precursor molecules as solids, PrL_3_, EuL_3_, and YbL_3_, were purchased from Sterm Chemicals, Inc. and used without further treatment. Each solid sample (i.e., handled separately) was slowly heated inside the vacuum chamber stepwise up to approximately 120–140 °C, slightly above the sublimation temperature, while pyrolysis was minimized, which normally occurs at much higher temperature above 200 °C. We assume that, only the precursor molecules (i.e., LnL_3_) can enter the gas phase for further laser ionization and fragmentation, while any metal-containing pyrolysis products, if they arise, would have stay in the solid phase because of their much higher sublimation temperature due to increased polarity upon the loss of ligands. The sample holder was home-made, consisting of a metal reservoir, which was wrapped with heating tape and attached to a thermocouple, allowing for both heating in vacuum and monitoring the temperature. The sample holder had a small opening (~0.5 mm) into a 0.5 mm diameter tube of 2 cm length pointing towards the focus of the laser lens. As the solid sample was transformed into the gas phase by heating, it effusively leaked through the tube and reached the laser focus for dissociative ionization and subsequent mass spectral (MS) detection. The distance between the tip of the tube and the laser focus could be adjusted for optimal gas density. The pressure of the vacuum chamber was 1~5 × 10^−6^ torr under effusive entry of the heated sample. Ion signals were collected and amplified with a 40 mm diameter microchannel plate, which was coupled to a digital oscilloscope (Lecroy 104MXi) for signal averaging and processing.

## Results and Discussion

This section presents our experimental results and combines the findings with prior long pulse studies^6,9,10,25^ to give an overall picture the multiple fragmentation pathways for LnL_3_.

**Figure 2:**
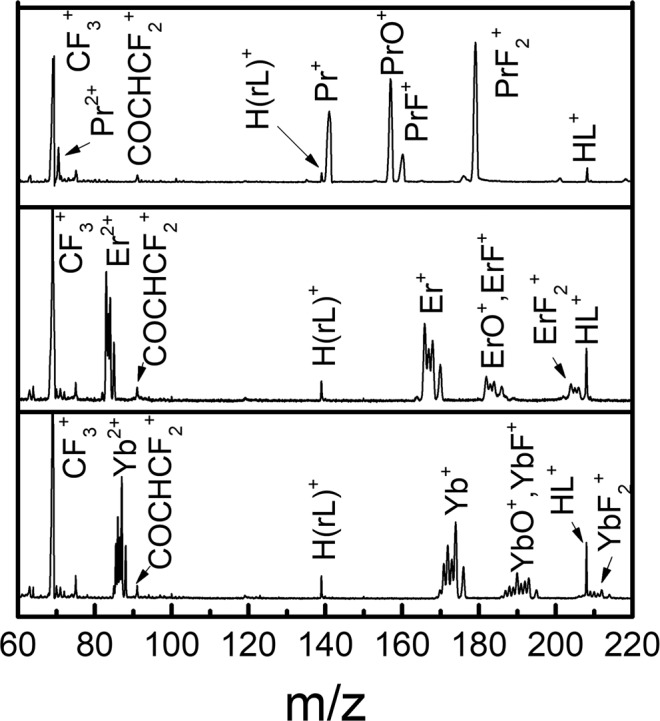
The low mass spectral region, from 60 to 220 Da, for PrL_3_ (top), ErL_3_ (middle), and YbL_3_ (bottom) generated with a TL pulse.

The gas-phase species PrL_3_, ErL_3_, and YbL_3_ were first photo-ionized and dissociated by the TL pulses. The TOF mass spectrum was recorded and calibrated using the abundant known masses as internal standards for linear fitting, which results in mass accuracy <1 Da across the entire relevant mass region. The low mass region of the measured photofragmentation mass spectra (60–220 Da) is shown in Fig. 2. The metal-containing peaks (e.g., LnF_2_^+^, LnF^+^, LnO^+^, Ln^+^ and Ln^2+^) are dominant in this region, where the mass resolving power of M/ΔM > 200 is sufficient to discern the isotopic peaks of Er and Yb that in each respective case are one mass unit apart. The peaks corresponding to non-metal-containing fragments, such as HL^+^, H(rL)^+^, CF_3_^+^, can also be seen in Fig. 2. The HL^+^ peak is often observed in mass spectrometry with LnL_3_ precursors, which may due to departing L radicals abstracting a H atom from neighboring ligands^9^. The CF_3_^+^ peak may arise from the ligand itself or from dissociation of metal-containing fragments. Another common peak at m/z = 91 in Fig. 2, is assigned to be COCHCF_2_^+^, an important by-product for understanding the fluoride-formation mechanism, which will be addressed below.

**Figure 3:**
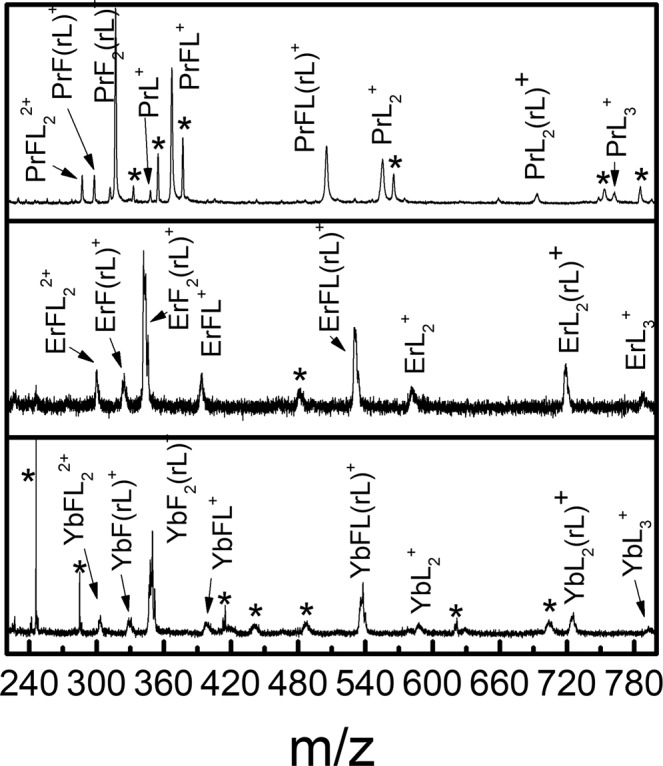
The high mass spectral region, from 220 to 800 Da, for PrL_3_ (top), ErL_3_ (middle), and YbL_3_ (bottom) generated with a TL pulse. The peaks labelled as * could not be uniquely assigned.

The high mass region of the spectra (220–800 Da) under TL pulse excitation is shown in Fig. 3. The mass resolving power of this region is estimated to be M/ΔM~150, allowing for reliable assignment of the majority of the peaks. Mass spectra for all the three lanthanide complexes considered here (i.e., PrL_3_, ErL_3_, and YbL_3_) share common types of most abundant photofragments: LnL_3_^+^, LnL_2_rL^+^, LnL_2_^+^, LnFL(rL)^+^, LnFL^+^, LnF_2_(rL)^+^, LnF(rL)^+^, and LnF(L)_2_^2+^. rL corresponds to CF_3_ loss from L, which is likely rearranged to a neutral ketene, O = C = CH-C(O)CF_3_^26^. The single ligand lanthanide fragment, LnL^+^, was only seen for PrL^+^. Many high mass fragments such as PrL^+^ have not been observed before in previous ns photofragmentation experiments^9,10,25^. In order to observe some high mass fragments for the hfac-related Lanthanide complexes under low intensity or long pulse laser excitation a polyether adduct had to be coordinated to the complexes^9^.

**Figure 4:**
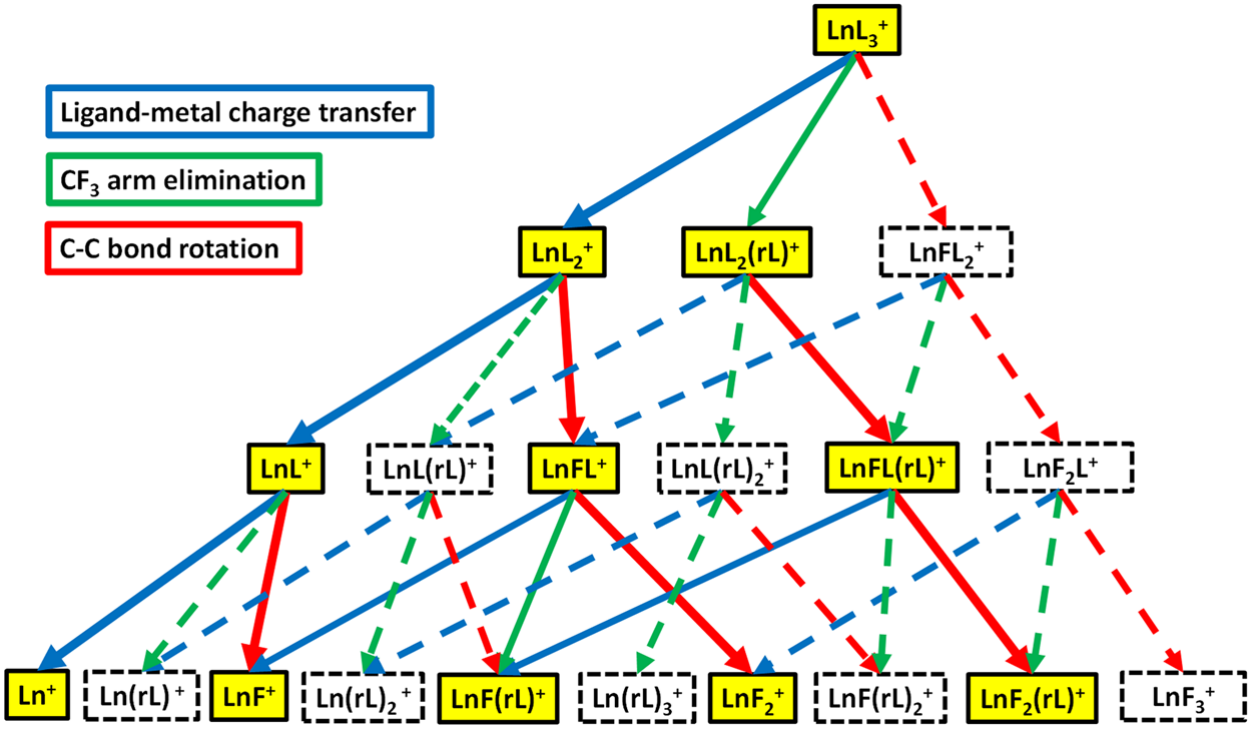
Proposed fluorinated-pathways during TL laser photofragmentation of LnL_3_. The blue, green, and red arrows correspond to ligand-metal charge transfer, CF_3_ elimination, and C-C bond rotation processes, respectively. The most abundant observed fragments are highlighted in yellow. The dashed rectangles correspond to fragments not observed during our experiments. The dashed arrows indicate the proposed paths connecting the non-observed photofragments.

In assigning the most abundant peaks observed in the mass spectra of Figs. 2 and 3, we observed that the Ln-containing fragment ion peaks are dominated by singly charged species. Additionally, there are only three types of moieties, (i.e., L, rL (see Fig. 1) and F, in various combinations), attached to Ln that appear in the mass spectrum. Observing that the number of bonded moieties did not exceed three, there is a total of 20 possible combinations of these moieties to form Ln-containing fragment ions, which cover most of the observed peaks in the mass spectra shown in Figs. 2 and 3. We summarize their m/z, assignment, along with the observed peak intensities in Table 1. Note that, for the m/z of Er or Yb containing fragment ions, the isotope averaged atomic weights are used. For peak intensities, we use ‘s’, ‘m’, ‘w’ to represent the peaks as having either ‘strong’, ‘medium’ or ‘weak’ signals, respectively, and chemically possible peaks with no signal are left as blank. Some additional fragments also shown in Figs. 2 and 3 are given in Table S1, as they do not appear to be directly related to the following proposed three main mechanistic pathways in Fig. 4: (a) ligand-metal charge transfer, (b) CF_3_ elimination, and (c) C-C bond rotation. There are also some peaks with unknown origin in Fig. 3 marked with ‘*’ that could not be assigned especially when attempting to include one Ln metal ion in their possible formulas. We expect that, these unknown substances are not relevant to the main features of the LnL_3_ fragmentation mechanism pathways in Fig. 4, which only involves Ln containing species.

**Table 1:** A list of the Ln (Ln = Pr, Er, Yb) species containing singly charged fragment ions, with ligands involving one or more of the following types of moieties: L, rL, and F.

PrL_3_			ErL_3_			YbL_3_			Number of moiety		
m/z	Assign.	sig.	m/z	Assign.	sig.	m/z	Assign.	sig.	L	rL	F
**762**	**PrL**_**3**_^**+**^	**w**	**788**	**ErL**_**3**_^**+**^	**w**	**794**	**YbL**_**3**_^**+**^	**w**	3	0	0
**555**	**PrL**_**2**_^**+**^	**m**	**581**	**ErL**_**2**_^**+**^	**w**	**587**	**YbL**_**2**_^**+**^	**w**	2	0	0
**693**	**PrL**_**2**_**(rL)**^**+**^	**w**	**719**	**ErL**_**2**_**(rL)**^**+**^	**w**	**725**	**YbL**_**2**_**(rL)**^**+**^	**w**	2	1	0
574	PrFL_2_^+^		600	ErFL_2_^+^		606	YbFL_2_^+^		2	0	1
**348**	**PrL**^**+**^	**w**	374	ErL^+^		380	YbL^+^		1	0	0
486	PrL(rL)^+^		512	ErL(rL)^+^		518	YbL(rL)^+^		1	1	0
**367**	**PrFL**^**+**^	**s**	**393**	**ErFL**^**+**^	**w**	**399**	**YbFL**^**+**^	**w**	1	0	1
624	PrL(rL)_2_^+^		650	ErL(rL)_2_^+^		656	YbL(rL)_2_^+^		1	2	0
**505**	**PrFL(rL)**^**+**^	**m**	**531**	**ErFL(rL)**^**+**^	**w**	**537**	**YbFL(rL)**^**+**^	**m**	1	1	1
386	PrF_2_L^+^		412	ErF_2_L^+^		418	YbF_2_L^+^		1	0	2
**141**	**Pr**^**+**^	**s**	**167**	**Er**^**+**^	**s**	**173**	**Yb**^**+**^	**s**	0	0	0
279	Pr(rL)^+^		305	Er(rL)^+^		311	Yb(rL)^+^		0	1	0
**160**	**PrF**^**+**^	**m**	**186**	**ErF**^**+**^	**m**	**192**	**YbF**^**+**^	m	0	0	1
417	Pr(rL)_2_^+^		443	Er(rL)_2_^+^		449	Yb(rL)_2_^+^		0	2	0
**298**	**PrF(rL)**^**+**^	**w**	**324**	**ErF(rL)**^**+**^	**w**	**330**	**YbF(rL)**^**+**^	w	0	1	1
555	Pr(rL)_3_^+^		581	Er(rL)_3_^+^		587	Yb(rL)_3_^+^		0	3	0
**179**	**PrF**_**2**_^**+**^	**s**	**205**	**ErF**_**2**_^**+**^	**m**	**211**	**YbF**_**2**_^**+**^	m	0	0	2
436	PrF(rL)_2_^+^		462	ErF(rL)_2_^+^		468	YbF(rL)_2_^+^		0	2	1
**317**	**PrF**_**2**_**(rL)**^**+**^	**s**	**343**	**ErF**_**2**_**(rL)**^**+**^	**m**	**349**	**YbF**_**2**_**(rL)**^**+**^	m	0	1	2
198	PrF_3_^+^		224	ErF_3_^+^		230	YbF_3_^+^		0	0	3

The number of ligands ranges from zero to three for each ion species. For each ion, the m/z, formula assignment and observed signal strength (‘s’, ‘m’ and ‘w’ respectively denoting strong, medium and weak) are shown. Those not observed are left blank in the sig. column.

Based on the photofragments observed in Figs. 1 and 2 under TL pulse excitation, we propose three competing dominant photofragmentation pathways leading to fluorinated products. The first one is ligand-metal charge transfer (LMCT), marked by the blue arrows in Fig. 3, which involves transferring an electron from one of the ligands to the metal center. A LMCT process is proposed to result in the sequential loss of a neutral ligand (i.e, LnL_3_^+^→LnL_2_^+^ + L^0^ →LnL^+^ + 2L^0^→Ln^+^ + 3L^0^). The intact neutral ligand likely leaves the metal center due to the loss of Coulomb attraction between the ligand and metal center. Being a radical with net charge equal to zero, the neutral ligand cannot be observed in the mass spectrometer. The observed metal-containing photofragments are likely due to post-ionization fragmentation. The LMCT photofragmentation process has been previously reported with ns pulses^9,26–28^. The proposed second dominant pathway involves the elimination of one CF_3_ from a ligand, and it is marked by the green arrows in Fig. 3. The occurrence of this process is evident by the presence of photofragments such as LnL_2_(rL)^+^ and LnF(rL)^+^ originating from their precursors LnL_3_^+^ and LnFL^+^, respectively. A large CF_3_^+^ peak can be seen in Fig. 1, and the elimination of CF_3_ from LnL_3_ and other hfac-coordinated transition metal complexes has also been observed in previous experiments^9,29–33^.

**Figure 5:**
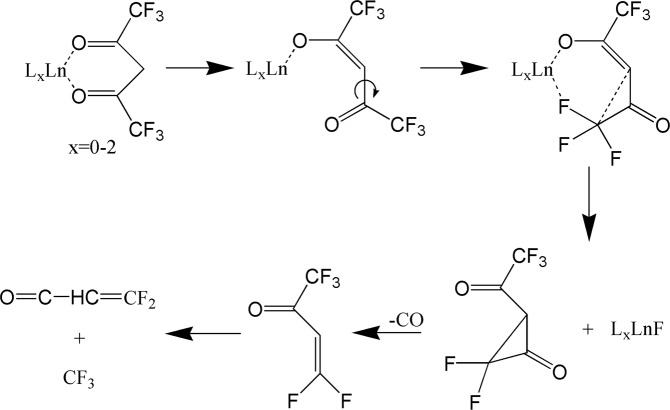
Illustration of Ln fluoride fragments resulting from C-C bond rotation according to refs. ^6,9–11^. The focus is in a single ligand with others possibly present as well.

The third pathway considered here consists of a C-C bond rotation process to form Ln fluorides. The detailed molecular structural changes linked to the C-C bond rotation process are illustrated in Fig. 4. This process has been proposed as commonly underlying the photo-fragmentation or thermo-fragmentation of organometallics with the hexafluoroacetylacetonate (hfac or L) ligand for generating metal fluorides, as previously reported by several research groups^6,9–11^. As shown in Fig. 5, the first step is believed to involve a metal-oxide bond elongation under laser field excitation, followed by the C_α_-C(O) bond rotation which brings the CF_3_ group into proximity to the metal. Afterwards, the metal fluoride forms along with CF_3_COCHCOCF_2_. The latter compound undergoes CO elimination and further bond arrangement to form COCHCF_2_^+^ (mass = 91) and CF_3_^+^ (mass = 69).

A significant contribution from the C-C bond rotation pathway is suggested by the observation of several Ln and fluorine containing compounds in Fig. 3, which were not observed during previous ns pulses experiments that only detected low mass spectra^9^. During the photofragmentation process, the formation of each new species involves the elimination of one ligand or the addition of a F atom to the Ln center from its precusor^9,10^. However, not all the expected fragments resulting from C-C bond rotation are present in our mass spectra, possibly due to their low stability.

From Table 1, as remarked earlier, the sum of the number of moieties L, rL and F in any observed ions is equal or less than three. This observation indicates the three main fragmentation processes of (1) LMCT, (2) CF_3_ elimination and (3) C-C rotation are competitive. The C-C bond rotation process is likely the dominant mechanism among these three competing processes, because most of the fragments that can arise as products of C-C bond rotation have very strong intensities (see Fig. 4 and Table 1), including LnL_2_(rL)^+^→LnFL(rL)^+^→LnF_2_(rL)^+^, LnL_2_^+^→LnFL^+^→LnF_2_^+^, and LnL^+^→LnF^+^. An exception to the preference for C-C bond rotation products appears to be associated with the non-observed fragments corresponding to the following channel: LnL_3_^+^→LnFL_2_^+^→LnF_2_L^+^→LnF_3_^+^. We expect that this finding is likely due to the sterically hindered LnL_3_^+^ with three ligands around Ln to restrict the C-C bond rotation in the first step (LnL_3_^+^→LnFL_2_^+^). The LMCT process should also be a significant mechanism because of the observed presence of most of its associated channels. A particularly strong LMCT-related channel present in our data is LnL_3_^+^→LnL_2_^+^→LnL^+^→Ln^+^. The CF_3_^+^ elimination process is likely the weakest amongst the three processes considered, as most fragments related to CF_3_^+^ elimination have not been seen. One interesting point is the clear occurrence of LnL_3_^+^→LnL_2_(rL)^+^. This may be related to the blocking of the LnL_3_^+^→LnFL_2_^+^ channel mentioned above which also leads to the enhancement of observed processes such as LnL_3_^+^→LnL_2_(rL)^+^ and LnL_3_^+^→LnL_2_^+^.

**Figure 6:**
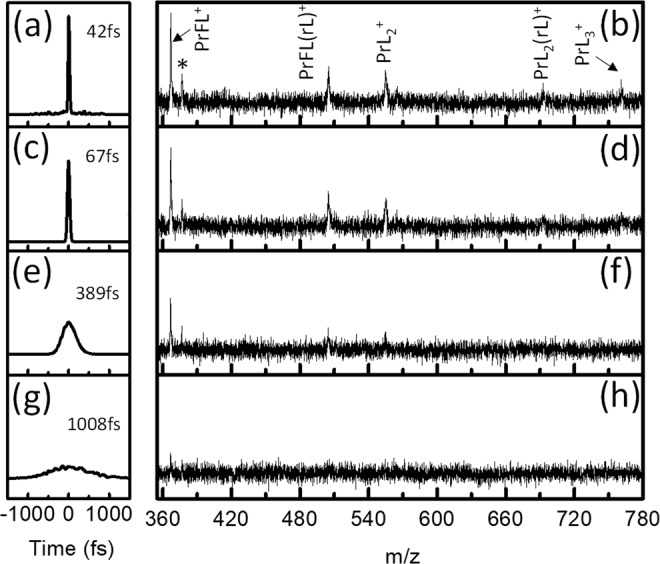
The linearly chirped laser pulses with different peak intensities, but with the same energy and different FWHM along with their corresponding mass spectra arising from PrL_3_. The FWHM of (**a,c,e,g**) are ~42 fs, ~67 fs, ~389 fs and ~1008 fs (the pulse shape plots are generated by the pulse simulations in the Lab2 software package developed by the Institute of Applied Physics in Bern, Switzerland), and their corresponding mass spectra of (**b,d,f,h**), respectively. The peaks labelled as * are either from a dimer or some unknown substance.

Figure 6 shows that four pulses with the same energy but different FWHM (i.e., generated by linear chirp with phase modulation using the pulse shaper) generate four mass spectra with different fragmentation patterns. The pulse with a FWHM of ~42 fs creates five identified high mass peaks, PrFL^+^, PrFL(rL)^+^, PrL_2_^+^, PrL_2_(rL)^+^, and PrL_3_^+^. As the pulse is chirped to FWHM = ~67 fs, the PrL_2_(rL)^+^, and PrL_3_^+^ fragment peaks disappear, and as the laser peak width is further elongated to FWHM = ~389 fs, fewer mass peaks are observed. For the broadest pulse with FWHM = ~1008 fs, the only high-mass peak left, PrFL^+^, can barely be seen over the background noise.

Thus, compressing the control pulse promotes production of larger amounts of high-mass fragments. Some aspects of this phenomenon can also be seen in Fig. 3. TL pulses (i.e., with FWHM of ~35 fs) generate the mass spectra with the largest content of high mass fragments, which provided crucial information towards deducing the overall mechanistic diagram shown in Fig. 4. The absence of high-intensity pulses may be the reason previous ns-laser studies could only observe small-mass products from LnL_3_ as the precursor, and thus only explicitly revealing a partial view of the overall dissociative-ionization mechanism.

In summary, Fig. 4 proposes an overall laser control mechanism mainly drawing from the current ultrafast experiments as well as insights obtained from previous long pulse dissociative-ionization studies. The latter studies specifically saw the species Ln^+^, LnF^+^, and LnF_2_^+^ in Fig. 4, but they did not reveal the intermediate species dynamics; however, these latter results are consistent with what revealed in the present experiments. The ultrafast experiments evidently can see the heavier ion precursor species lost in the longer pulse experiments. It is noteworthy that experiments on such disparate time scales can be joined together in an appropriated fashion for a proposed overall mechanistic picture. Figure 4 still has unobserved species, many of which may be unstable calling for other ultrafast optical spectroscopic probe experiment to observe. Additionally, Table 1 contains some species whose connection to the present mechanism are left unexplained.

## Conclusion

This paper presents the results of a series of experiments for the dissociative-ionization of LnL_3_ driven by both TL and linearly chirped ultrafast fs laser pulses. Various fluorine and Ln containing high mass fragments were observed for the first time through the use of a fs laser source leading to new evidence for a general understanding of the photo-dissociation mechanism. The high-mass fragments obtained from TL pulse excitation provide insights that suggest a set of consistent fragmentation pathways. Our experimental results may be interpreted in terms of photofragmentation and fluoride formation in a stepwise mechanism. An overall proposed mechanism consists of three main distinct components: (a) ligand-metal charge transfer, (b) CF_3_ elimination, and (c) C-C bond rotation processes. Previous ns studies were only able to resolve part of the full mechanistic picture due to missing information on the high mass fragments, which are proposed here as forming the early stages of the mechanism. The laser pulse experiments with decreasing intensity but longer pulse length support the prior findings that high mass fragments from LnL_3_ were not observed in ns or longer pulse experiments. Finally, we remark that quantum chemistry dynamics calculations of suitable quality, including the tailored laser pulses, would be valuable to compliment the experimental work reported in this paper.

## Supplementary information

Supplementary Information.
