# Supplementary material for: Ultrafast Photofragmentation of Ln(hfac)3 with a Proposed Mechanism for forming High Mass Fluorinated Products
Source: Sci Rep. 2020 Apr 27;10:7066. doi: 10.1038/s41598-020-64015-2 (PMC7184609; doi:10.1038/s41598-020-64015-2)
Supplement: Supplementary file 1 — Supplementary Information. [file 41598_2020_64015_MOESM1_ESM.docx]

Supporting Information

Ultrafast Photofragmentation of Ln(hfac)_3_ with a Proposed Mechanism for forming High Mass Fluorinated Products

Jiangchao Chen, Xi Xing, Roberto Rey-de-Castro, and Herschel Rabitz*

*Department of Chemistry, Princeton University, Princeton, New Jersey 08544, USA*

* To whom correspondence should be addressed. E-mail: [hrabitz@princeton.edu](mailto:hrabitz@princeton.edu)

**Figure S1:** Mass spectra for PrFL_2_^2+^ (top), ErFL_2_^2+^ (middle), and YbFL_2_^2+^ (bottom) after TL pulse excitation. The blue lines and ticks indicates Er and Yb isotope variants.

Figure S1 displays detailed views of the mass peaks for PrFL_2_^2+^, ErFL_2_^2+^ and YbFL_2_^2+^, centered at m/z=287, 300 and 303, respectively. ErFL_2_^2+^ and YbFL_2_^2+^ have broadened peak shapes due to isotope effects. The location of the isotopic peaks are marked on top of each spectrum (blue lines and ticks) in Figure S1. All the peak patterns and precise peak positions clearly indicate that LnFL_2_^2+^ is transiently present in all these cases. The formation of LnFL_2_^2+^ requires either a Ln(V) or a biradical, which are very unstable, or, a structure of the form LnL(FL)^2+^, where the departing ligand (the L radical) left an F radical on the retained L and then metal oxidation to the state Ln(III). As FL is neutral, that would mean the LnL(FL)^2+^ came from LnL_3_^2+^, which would likely be highly unstable with two of the ligands as radicals loosely coordinated to an Ln(III) metal ion. The absense of LnFL_2_^+^ in our detected mass spectra cannot be interpreted as it being further ionized to form the observed LnFL_2_^2+^. Additional work is needed to better understand the LnFL_2_^2+^ formation pathway.

**Table S1:** Other fragments are also observed in Figures 1 and 2, but not presented in Figure 3, because they do not appear to be directly related the proposed three pathways: (a) ligand-metal charge transfer, (b) CF_3_ elimination, and (c) C-C bond rotation processes. For each ion, the m/z, formula assignment and observed signal strength (‘s’, ‘m’ and ‘w’ respectively denoting strong, medium and weak) are shown.

| **Other Fragments** | | | | | | | | |
| --- | --- | --- | --- | --- | --- | --- | --- | --- |
| **PrL_3_** | | | **ErL_3_** | | | **YbL_3_** | | |
| **m/z** | **Assign.** | **Int.** | **m/z** | **Assign.** | **Int.** | **m/z** | **Assign.** | **Int.** |
| 287 | PrFL_2_^2+^ | w | 300 | ErFL_2_^2+^ | w | 303 | YbFL_2_^2+^ | w |
| 208 | HL^+^ | w | 208 | HL^+^ | w | 208 | HL^+^ | w |
| 157 | PrO^+^ | s | 183 | ErO^+^ | m | 189 | YbO^+^ | m |
| 139 | H(rL)^+^ | w | 139 | H(rL)^+^ | w | 139 | H(rL)^+^ | w |
| 91 | COCHCF_2_^+^ | w | 91 | COCHCF_2_^+^ | w | 91 | COCHCF_2_^+^ | w |
| 70.5 | Pr^2+^ | w | 83.5 | Er^2+^ | s | 86.5 | Yb^2+^ | s |
| 69 | CF_3_^+^ | s | 69 | CF_3_^+^ | s | 69 | CF_3_^+^ | s |
